# Case Report: Rabies Vaccine-Induced Thrombotic Thrombocytopenic Purpura in a Patient With Systemic Lupus Erythematosus

**DOI:** 10.3389/fimmu.2022.851316

**Published:** 2022-04-25

**Authors:** Yanming Cui, Jianbo Wei, Xiang Peng

**Affiliations:** Department of Rheumatology and Immunology, The Sixth Affiliated Hospital of Guangzhou Medical University, Qingyuan People’s Hospital, Guangdong, China

**Keywords:** vaccine, thrombotic thrombocytopenic purpura, systemic lupus erythematosus, autoimmune disease, ADAMTS13

## Abstract

For patients with autoimmune diseases, vaccination is controversial. The use of vaccination in patients with autoimmune diseases is controversial. There are many reports of secondary thrombotic thrombocytopenic purpura cases after various vaccinations. Thrombotic thrombocytopenic purpura is a rare thrombotic microangiopathy characterized by microvascular pathological hemolytic anemia, severe thrombocytopenia, and ischemic organ damage with a very high fatality rate. We report a case of thrombotic thrombocytopenic purpura in a patient with systemic lupus erythematosus after rabies vaccination. She developed gastrointestinal bleeding nearly a month after the vaccination. Laboratory tests confirmed a severe deficiency of ADAMTS13 and the presence of ADAMTS13 autoantibodies. Through early identification of thrombotic thrombocytopenic purpura, immunosuppressive therapy, and plasma exchange treatment, the patient was saved from danger. This case suggests that attenuated vaccines may also have unexpected adverse effects in patients with long-term use of immunosuppressive drugs and autoimmune diseases. To our knowledge, this is the first case report of thrombotic thrombocytopenic purpura in a patient with systemic lupus erythematosus secondary to rabies vaccination.

## Introduction

In general, vaccination is safe and the benefits far outweigh the risks. However, even in healthy people, adverse reactions may occur after vaccination, such as reactive arthritis, immune thrombocytopenia, and Guillain–Barre syndrome (1). The human rabies vaccines currently approved for marketing in China include Vero cell purified vaccine, HDCV, hamster kidney primary cell purified vaccine, and primary chicken embryo cell purified vaccine. The World Health Organization (WHO)-recommended vaccination schedule is the “5-shot method” after exposure, that is, one injection each at 0, 3, 7, 14, and 28 days after exposure. In particular, it is recommended that patients receiving immunosuppressive therapy (including antimalarial drugs) should be given a double dose of the first injection. Although many studies have confirmed the safety of rabies vaccines, adverse reaction is still common, including injection site pain, headache, nausea, allergies, fever, and fatigue (2). Thrombotic thrombocytopenic purpura (TTP) is a rare vaccine-related adverse reaction. As of 2021, we searched the Centers for Disease Control and Prevention (CDC) WONDER for data on adverse reaction of rabies vaccines, and found that thrombocytopenia accounted for 0.17%, and thrombocytopenic purpura accounted for 0.02% [Vaccine Adverse Event Reporting System (VAERS) using CDC WONDER Online Database; available from https://wonder.cdc.gov/vaers.html]. To our knowledge, only one case of rabies vaccine-related TTP was reported in 2014 in a 28-year-old healthy man (3), and this case study was the first to report a systemic lupus erythematosus (SLE) patient who was diagnosed with TTP after rabies vaccination. Interestingly, this patient is using immunosuppressive therapy regularly and is in stable condition.

## Case Presentation

A 49-year-old woman was diagnosed with systemic lupus erythematosus and lupus nephritis [focal proliferative erythematosus nephritis with thickening of glomerular basement membrane, III (A+) + V type]; the condition was stabilized after treatment with methylprednisolone, cyclophosphamide (accumulated 7.2 g), hydroxychloroquine, and so on. The dosage of immunosuppressive agents was standardized and adjusted. Due to stable disease control, hormones were discontinued from January 2018. At the same time, hydroxychloroquine 0.1 g bid and methotrexate 10 mg qw were given for long-term maintenance treatment. From May 2020, she was given mycophenolate mofetil 0.25 mg bid and hydroxychloroquine 0.2 g bid, and regular outpatient follow-ups. On April 1, 2021, the patient was followed up and laboratory indices were detected—hemoglobin (HB) was 123 g/L, platelets (PLT) were 150×10^9^/L, white blood cells were 4.53×10^9^/L, urine protein and urine occult blood were all negative, and complement C3 and C4 were normal. The results of the examination showed that the lupus condition was stable. After the fourth vaccination, the patient developed dizziness and fatigue. On May 10, 2021, the patient began to pass black stool and came to the hospital again on May 13. At that time, the patient had no symptoms of lupus activity such as fever, new skin rash, oral ulcers, and obvious hair loss, and her blood pressure was under control recently. Questioning the patient’s medical history, the patient was scratched by a cat on her left foot on March 28, and was vaccinated with a freeze-dried human rabies vaccine (Ningbo Rongan Biological Manufacturing Co., Ltd., drug batch number: National Medicine Standard S20073014) on March 28, April 4, April 6, and April 12, a total of 4 times.

**Figure 1:**
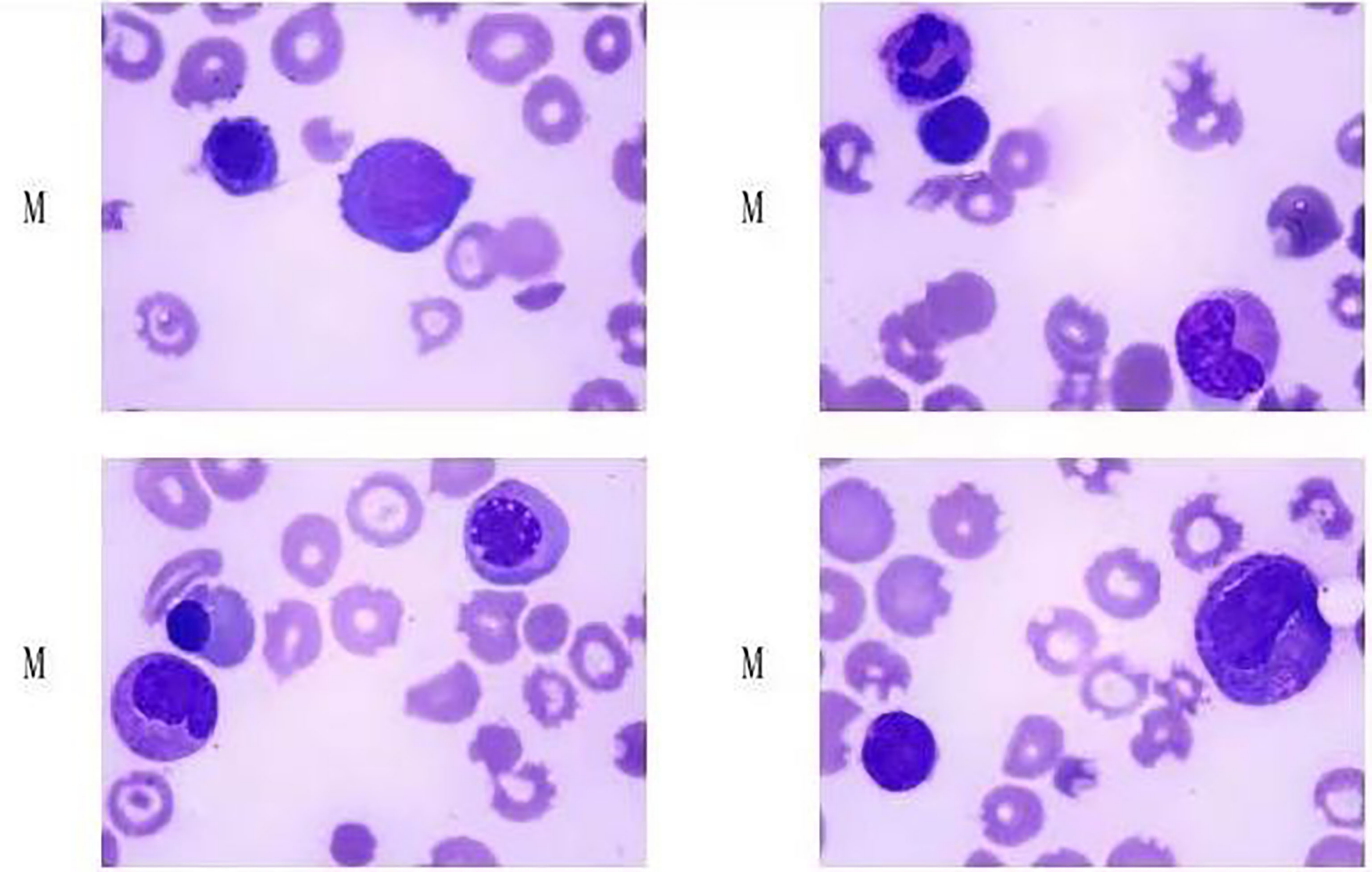
Patient’s bone marrow smear. (Hyperplasia was active in bone marrow, no obvious compensatory hyperplasia and thrombocytopenia were observed in red system.)

**Figure 2:**
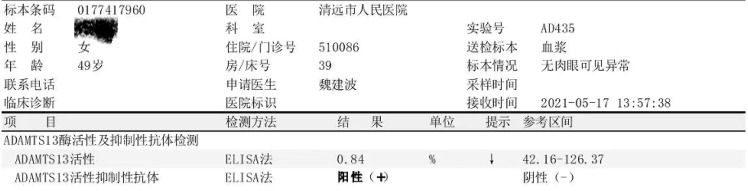
The result of ADAMTS13 autoantibodies and ADAMTS13 inhibitory autoantibodies.

On admission, the patient’s physical examination showed stable vital signs, anemia, yellow sclera, mild upper abdomen tenderness, subcutaneous bleeding spots and ecchymosis on the limbs, and no obvious abnormalities in the other systemic examinations. Laboratory examination revealed that hemoglobin 46 g/L, platelets 4×10^9^/L, white blood cells 3.65×10^9^/L, reticulocyte count 11.62%, stool OB (+), urine protein (3+), urine occult blood (4+), D-Dimer 2.99 mg/L, and coagulation function were normal; creatinine 39.2 μmol/L, aspartate aminotransferase (AST) 58U/L, urea, albumin (ALB), and alanine aminotransferase (ALT) were normal; total bilirubin 43.7 μmol/L, indirect bilirubin 30.3 μmol/L, direct bilirubin 13.4 μmol/L, lactate dehydrogenase (LDH) 1,166 U/L; Coombs test (-); Broken red blood cells can indeed be seen on peripheral blood smears; ANA 1:640 granular type, lupus anticoagulant, and anticardiolipin antibody antibodies were negative; complement C3, C4, and anti-dsDNA were normal; G-6-PD was normal; bone marrow smear showed active bone marrow hyperplasia, no obvious compensatory hyperplasia, and thrombocytopenia in the erythroid (**Figure 1**); no paroxysmal nocturnal hemoglobinuria (PNH) clones were detected in the bone marrow, and no immunophenotypic abnormalities related to acute leukemia and high-risk MDS were detected; infection-related tests were negative. She was initially diagnosed with rabies vaccination-related thrombotic thrombocytopenic purpura based on clinical manifestations and examinations, ruling out other possible causative factors. On the 7th day of admission, the result of ADAMTS13 autoantibodies (ELISA assay) were 0.84%, and ADAMTS13 inhibitory autoantibodies were positive, confirming our diagnosis (**Figure 2**).

**Figure 3:**
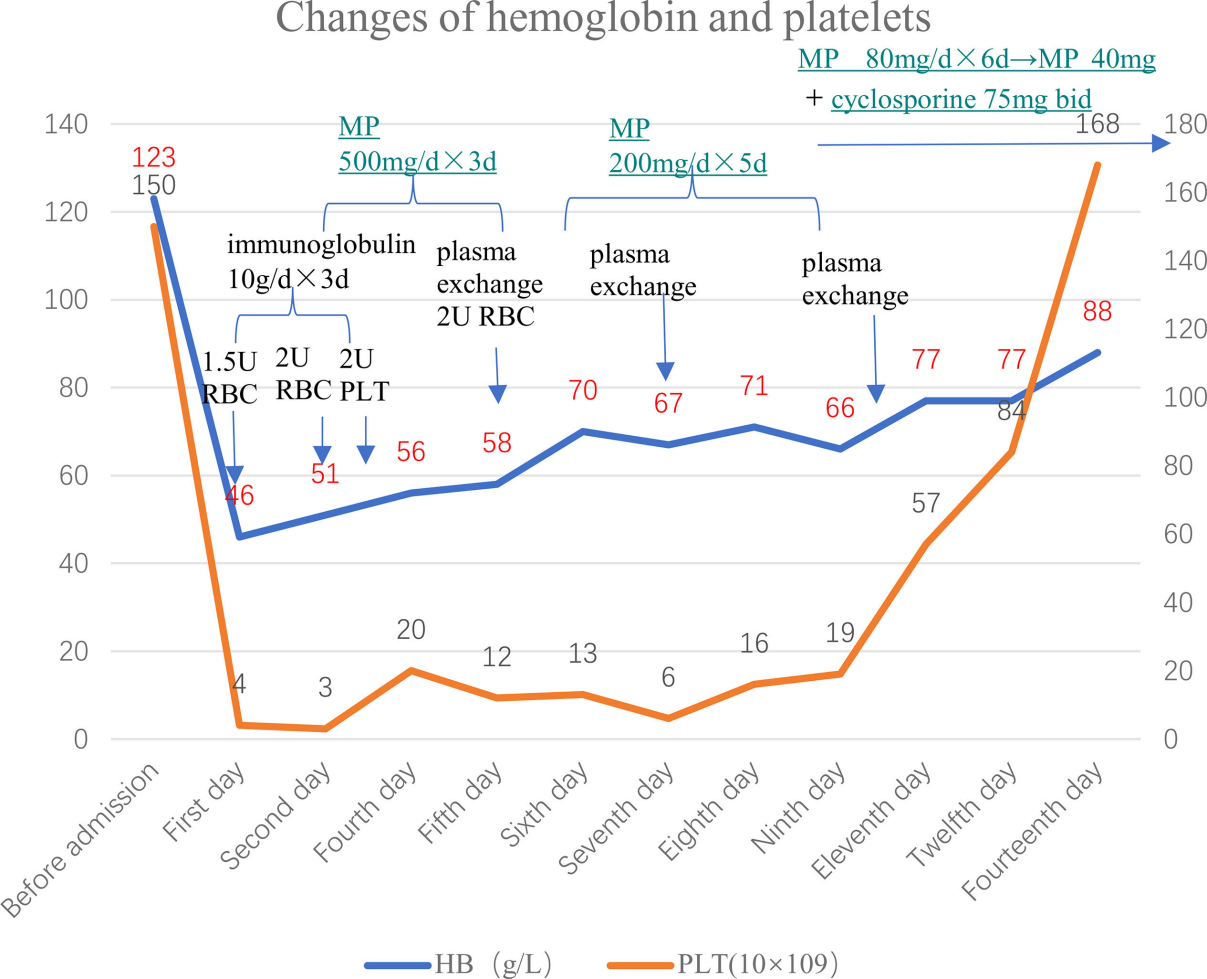
Changes of hemoglobin and platelets during treatment.

**Figure 4:**
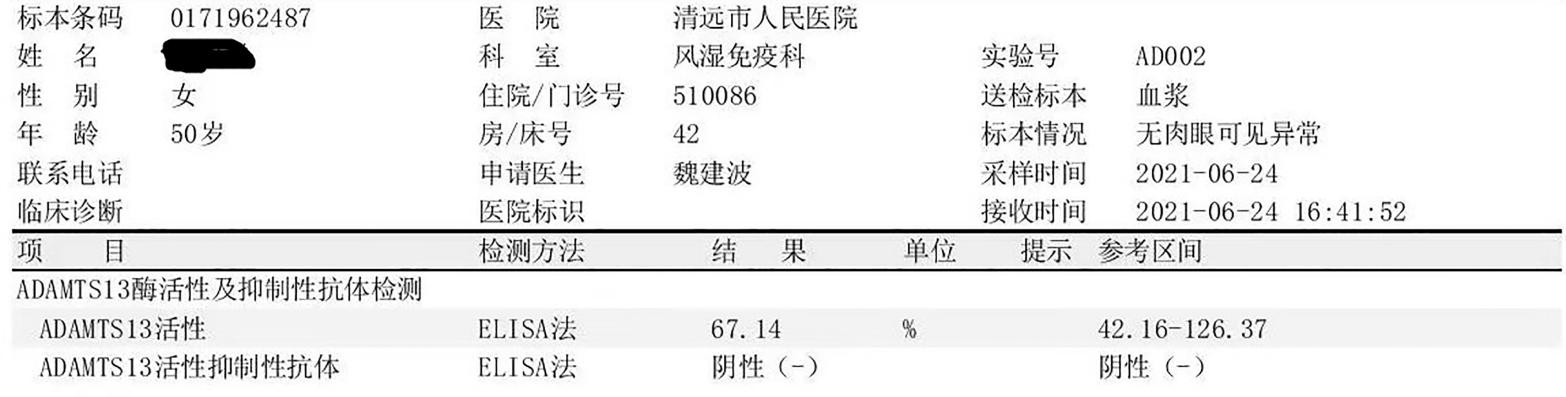
The result of ADAMTS13 autoantibodies and ADAMTS13 inhibitory autoantibodies after treatment.

On the first day of admission, the patient received 1.5 U red blood cell (RBC) and immunoglobulin therapy (10 g per day for 3 days). On the second day, the patient received 2 U RBC and started receiving high-dose methylprednisolone (MP) therapy (500 mg per day for 3 days, then 200 mg per day for 5 days), followed by methylprednisolone 80 mg/day, and changed to 40 mg after 6 days; On the third day of admission, the patient received 2 U of PLT. On the fifth day of admission, the patient received 1.5 U RBC again, and plasma exchange treatment was initiated at the same time [a total of 3 times, on the fifth day, the seventh day, and the tenth day; each time about 1,500–2,000 ml type O RhD(+) plasma with the treatment mode of single filtration plasmapheresis for a duration of nearly 4 h]. On the eleventh day of admission, hemoglobin (77 g/L) and platelets (57×10^9^/L) were both higher than before, while total bilirubin (13.9 μmol/L) and indirect bilirubin (7.1 μmol/L), direct bilirubin (6.8 μmol/L), and LDH (264 U/L) were all lower than before. On the fourteenth day of admission, hemoglobin had risen to 88 g/L, and platelets had returned to normal. The patient was treated with methylprednisolone 40 mg qd and cyclosporine 75 mg bid when she was discharged, and the dose of methylprednisolone was reduced regularly. The above treatment process is shown in **Figure 3**. During follow-up in the past month, the patient’s platelets remained stable and hemoglobin returned to baseline 123 g/L. After a month, the ADAMTS13 autoantibodies rose to 67.14%, and the ADAMTS13 inhibitory autoantibodies were negative (**Figure 4**).

## Discussion

Thrombotic thrombocytopenic purpura is a rare life-threatening thrombotic microangiopathy characterized by microvascular pathological hemolytic anemia, severe thrombocytopenia, and ischemic organ damage, which can lead to multiple organ failure and ultimately lead to death with missed diagnosis and untimely treatment.

The lack of ADAMTS13 plays a crucial role in the pathogenesis of thrombotic thrombocytopenic purpura. TTP can be diagnosed when the activity of ADAMTS13 is less than 10% (4). According to the lack of mechanism of ADAMTS13, TTP is divided into congenital and acquired. Congenital TTP is due to persistent ADAMTS13 deficiency caused by biallelic ADAMTS13 gene, while acquired TTP is due to ADAMTS13 deficiency mediated by autoantibodies (5). In our case, the ADAMTS13 activity of the patient is significantly decreased and the autoantibody is positive. After treatment, the ADAMTS13 activity can be restored to normal and the autoantibody turned negative, showing that it is an acquired TTP.

Although most cases are idiopathic, there may be different triggers, such as bacterial or viral infection (such as HIV infection), immune disease, malignant tumor, pregnancy, and drugs (4–6). However, in clinical practice, there are also many reports about secondary TTP after vaccination (7–10). As far as current research is concerned, the pathogenesis of vaccine-related TTP is still unclear. The main causal judgment is that there are no other potential causes, and there is an obvious time correlation. The patient in this case developed TTP nearly a month after vaccination with rabies, which is longer than the onset time reported in a previous case (3). Although TTP can be secondary to systemic lupus erythematosus, the relevant examination indicators in the outpatient clinic before the onset indicate that the lupus condition is in a stable stage. Therefore, TTP caused by lupus is not considered. Typical TTP includes fever, microangiopathic hemolytic anemia, thrombocytopenia, central nervous system involvement, and renal insufficiency. However, most patients do not all have the above-mentioned “five signs” (11). The patient in this case developed severe thrombocytopenia and microangiopathic hemolytic anemia after rabies vaccination that could not be explained by the original disease (red blood cell fragments were seen on blood smears, total bilirubin increased, indirect bilirubin increased mainly, LDH is elevated, and Coombs test is negative), and the patient developed neurological symptoms (manifested as headache) during the treatment. At that time, the head CT showed no abnormalities. Therefore, we are highly suspicious of TTP and timely start glucocorticoid pulse therapy and plasma exchange therapy, after which the patient finally improved and the level of ADAMTS13 finally confirmed our diagnosis. The patient had bleeding at the beginning and the platelet count was extremely low, so 2 U platelets were given. We stopped the platelet transfusion soon after we realized it might be TTP, because it is an absolute contraindication to give platelets to TTP patients.

There are currently no studies evaluating the safety and immunogenicity of rabies vaccination in SLE patients. The use of DMARDs (such as chloroquine) or immunosuppressive agents including corticosteroids may weaken the immune response to the vaccine, but overall, inactivated vaccines seem to be safe for SLE patients (12). However, the patient in this case developed a rare TTP after following an inactivated rabies vaccine. Therefore, the safety and effectiveness of vaccination for SLE patients who are on immunosuppressive therapy or treatment patients need to be further studied. In view of this experience, TTP should be considered in the differential diagnosis of patient with thrombocytopenia and anemia after vaccination.

## Data Availability Statement

The original contributions presented in the study are included in the article/supplementary material. Further inquiries can be directed to the corresponding author.

## Ethics Statement

The patient and next of kin provided written informed consent for the publication of this case report.

## Author Contributions

XP, YC, and JW conceptualized the work. YC and JW drafted the manuscript. XP performed the investigations and critically revised the manuscript. All authors contributed to the article and approved the submitted version.

## Conflict of Interest

The authors declare that the research was conducted in the absence of any commercial or financial relationships that could be construed as a potential conflict of interest.

## Publisher’s Note

All claims expressed in this article are solely those of the authors and do not necessarily represent those of their affiliated organizations, or those of the publisher, the editors and the reviewers. Any product that may be evaluated in this article, or claim that may be made by its manufacturer, is not guaranteed or endorsed by the publisher.

## References

[1] Ruhrman-Shahar N Torres-Ruiz J Rotman-Pikielny P Levy Y. Autoimmune Reaction After Anti-Tetanus Vaccination—Description of Four Cases and Review of the Literature. Immunol Res (2017) 65:157–63. doi: 10.1007/s12026-016-8822-x 27435706

[2] Wang L Sun M Zhang X. Adverse Reaction and Immunogenicity of Domestic Freeze-Dried Rabies Vac Cine(Vero Cells)for Human Use. Chin J Biologicals (2008) 21:1115–7. doi: 10.3696/j.issn.1004-5503.2008.12.024

[3] Kadikoylu G Yavasoglu I Bolaman Z. Rabies Vaccine-Associated Thrombotic Thrombocytopenic Purpura. Transfus Med (2014) 24:428–9. doi: 10.1111/tme.12160 25388930

[4] Sukumar S Lämmle B Cataland SR. Thrombotic Thrombocytopenic Purpura: Pathophysiology, Diagnosis, and Management. J Clin Med (2021) 10:536. doi: 10.3390/jcm10030536 33540569 PMC7867179

[5] Schultz NH Sørvoll IH Michelsen AE Munthe LA Lund-Johansen F Ahlen MT. Thrombosis and Thrombocytopenia After ChAdOx1 Ncov-19 Vaccination. N Engl J Med (2021) 384:2124–30. doi: 10.1056/NEJMoa2104882 PMC8112568 33835768

[6] Scully M Blombery P. Management of Thrombotic Thrombocytopenic Purpura: Current Perspectives. J Blood Med (2014) 5:15–23. doi: 10.2147/JBM.S46458 24523598 PMC3921093

[7] Maayan H Kirgner I Gutwein O Herzog Tzarfati K Rahimi Levene N Koren Michowitz M. Acquired Thrombotic Thrombocytopenic Purpura: A Rare Disease Associated With BNT162b2 Vaccine. J Thromb Haemost (2021) 19:2314–17. doi: 10.1111/jth.15420 PMC8237075 34105247

[8] Yocum A Simon E. Thrombotic Thrombocytopenic Purpura After Ad26.COV2-S Vaccination. Am J Emerg Med (2021) 49:441.e3–4. doi: 10.1016/j.ajem.2021.05.001 PMC8095021 33980419

[9] de Bruijn S Maes MB De Waele L Vanhoorelbeke K Gadisseur A. First Report of a *De Novo* iTTP Episode Associated With an mRNA-Based Anti-COVID-19 Vaccination. J Thromb Haemost (2021) 19:2014–18. doi: 10.1111/jth.15418 PMC8236927 34105244

[10] Yavaşoğlu 0. Vaccination and Thrombotic Thrombocytopenic Purpura. Turk J Hematol (2020) 37:218–9. doi: 10.4274/tjh.galenos.2020.2020.0060 PMC7463209 32227797

[11] Scully M Yarranton H Liesner R Cavenagh J Hunt B Benjamin S. Regional UK TTP Registry: Correlation With Laboratory ADAMTS 13 Analysis and Clinical Features. Brit J Haematol (2008) 142:819–26. doi: 10.1111/j.1365-2141.2008.07276.x 18637802

[12] Garg M Mufti N Palmore TN Hasni SA. Recommendations and Barriers to Vaccination in Systemic Lupus Erythematosus. Autoimmun Rev (2018) 17:990–1001. 30103044 10.1016/j.autrev.2018.04.006 PMC6150817

